# Local radiotherapeutic management of ependymomas with fractionated stereotactic radiotherapy (FSRT)

**DOI:** 10.1186/1471-2407-6-222

**Published:** 2006-09-07

**Authors:** Stephanie E Combs, Christoph Thilmann, Jürgen Debus, Daniela Schulz-Ertner

**Affiliations:** 1 Department of Radiation Oncology, University of Heidelberg, INF 400, 69120 Heidelberg, Germany

## Abstract

**Background:**

To assess the role of Fractionated Stereotactic Radiotherapy (FSRT) in the management of ependymomas.

**Methods:**

From January 1992 to July 2003, FSRT was performed in 19 patients with histologically confirmed ependymomas. The median age was 15 years, 5 patients were younger than 4 years of age.

Twelve patients received FSRT as primary postoperative radiotherapy after surgical resection. In 6 patients irradiation of the posterior fossa was performed with a local boost to the tumor bed, and in 4 patients the tumor bed only was irradiated. In 7 patients FSRT was performed as re-irradiation for tumor progression. This patient group was analyzed separately. A median dose of 54 Gy was prescribed in a median fractionation of 5 × 1.8 Gy per week for primary RT using 6 MeV photons with a linear accelerator. For FSRT as re-irradiation, a median dose of 36 Gy was applied. All recurrent tumors were localized within the former RT-field.

**Results:**

The 5- and 10-year overall survival rates were 77% and 64%, respectively. Patients treated with FSRT for primary irradiation showed an overall survival of 100% and 78% at 3 and 5 years after irradiation of the posterior fossa with a boost to the tumor bed, and a survival rate of 100% at 5 years with RT of the tumor bed only. After re-irradiation with FSRT, survival rates of 83% and 50% at 3-and 5 years, respectively, were obtained.

Progression-free survival rates after primary RT as compared to re-irradiation were 64% and 60% at 5 years, respectively.

FSRT was well tolerated by all patients and could be completed without interruptions due to side effects. No severe treatment related toxicity > CTC grade 2 for patients treated with FSRT could be observed.

**Conclusion:**

The present analysis shows that FSRT is well tolerated and highly effective in the management of ependymal tumors. The rate of recurrences, especially at the field border, is not increased as compared to conventional radiotherapy consisting of craniospinal irradiation and a local boost to the posterior fossa.

## Background

Ependymomas (EP) originate from ependymal cells of the wall of the cerebral ventricles, the central canal of the spinal cord, and from ependymal remnants in the filum terminale, the choroid plexus or the white matter adjacent to the highly angulated ventricular surface [1]. Additionally, ependymal neoplasms can be caused by the migration of fetal ependymal cell residuals from periventricular areas into the brain parenchyma [2].

The group accounts for 3% of all intracranial neoplasms, and they are the third most common tumor diagnosed in children. About 10% of childhood central nervous system tumors are ependymomas (EPs), and 50% of patients are younger than 3 years of age [3-5]. Most ependymal tumors arise infratentorially, commonly in the area of the fourth ventricle; about 10–15% arise along the spinal axis [3-7]. EPs constitute 3–9% of all neuroepithelial tumors, and 50–60% of spinal gliomas [1,3,8-12].

The World Health Organization (WHO) classification of central nervous system tumors recognizes various groups of EP: Subependymomas (WHO Grade I), ependymomas (WHO Grade II), and anaplastic ependymomas (WHO Grade III) [8]. The biological activity of EP is related to the intracranial location; infratentorial tumors show a higher mitotic activity than supratentorial tumors [13].

The management of ependymal tumors is discussed controversially. Commonly, multimodality treatment including surgery and radiotherapy is performed. Research is focusing on precision RT techniques enabling local dose escalation. In the past, craniospinal irradiation (CSI) was recommended to be the standard radiotherapeutic approach. Bloom et al. recommend CSI especially for high grade tumors localized infratentorially [14]. However, more and more studies have shown that localized irradiation of the posterior fossa or even volumes as small as the tumor bed and a 1.5–2 cm safety margin are equally effective [12,24,34,49]. As a positive dose-effect-relationship with regard to the prescribed RT dose is known for EP [15-17], high precision radiotherapy techniques such as fractionated stereotactic radiotherapy (FSRT) might be beneficial in the treatment of patients with EP since high local doses can be applied to a defined target volume while sparing surrounding normal tissues. However, the role of FSRT is not yet clearly defined in the treatment of EP. The present study reports our results of locoregional RT for patients with EP using FSRT, analyzes prognostic factors and reflects results and recommendations found in the literature.

## Methods

### Study population

From January 1992 to December 2003, 17 patients with histologically confirmed EP were treated with FSRT.

The data presented in this analysis were acquired retrospectively and anonymously; therefore, no official approval by the local ethics committee was necessary.

All patients had received a complete work-up after primary diagnosis, including MRI of the brain, spine as well as a lumbar puncture with craniospinal fluid cytology to allow for exact staging. The degree of surgical resection was based on the neurosurgeon's opinion as well as on postoperative MRI-scans. Data on patients' age, date of diagnosis, treatment performed, including surgery, radiotherapy as well as chemotherapy were collected. With regard to RT, irradiated volumes, radiation doses, treatment failures, second-line treatment and survival were documented. Histological diagnoses were all reviewed and categorized according to the World Health Organization classification [18].

### Radiotherapy treatment planning for FSRT

As described previously, contrast-enhanced CT and MRI scans were performed in an individual Scotch cast^® ^Mask fixation for three dimensional treatment planning [19,20]. The target volume was defined after image correlation of contrast enhanced CT and MR-images with a slice thickness of 3 mm, using the Voxelplan^® ^software developed at the German Cancer Research Center [21,22]. Median size of PTV for FSRT was 17.8 ml (range 4.5–281 ml), consisting of the area of contrast enhancement in T1-weighted MRI-sequences, adding a 1–2 cm safety margin to include microscopic spread of tumor cells.

All patients gave written informed consent to the treatment.

### Follow-up

Patients were seen for follow-up visits 6 weeks after completion of radiotherapy, then in regular 3 to 6 months intervals depending on the clinical condition of the patient. Follow-up visits included a contrast-enhanced MRI as well as a complete clinical examination including a thorough neurological assessment. Additional MRI scans and clinical assessment were performed if new symptoms developed or deterioration was observed. Whenever tumor progression and radiation induced necrosis could not be distinguished by MR-imaging, additional PET-examinations and MR-spectroscopy were scheduled. Side effects were documented according to the Common Toxicity Criteria (CTCAE) Version 3.0.

### Statistics

Overall survival was calculated from the time point of primary diagnosis of ependymoma; survival after re-irradiation was calculated from initiation of FSRT. Progression-free survival after re-irradiation was calculated from the initiation of re-irradiation treatment until tumor progression or death (by any cause), whichever happened first, using the Kaplan Meier method [23]. The potential prognostic factors age, RT technique, grade, location and extent of surgical resection were analyzed for the group of primary localized tumors, using the univariate Cox proportional regression model. Statistical analyses were performed using the software program Statistica 6.0 (StatSoft, Hamburg, Germany).

## Results

### Patient characteristics

From October 1989 to December 2004, 19 patients with histologically diagnosed EPs were treated with Fractionated Stereotactic Radiotherapy (FSRT).

Patient's characteristics are summarized in Table 1. The median age at primary diagnosis was 15 years (range 1–60 years). Five patients were younger than 4 years of age. The male:female ratio was 10:9.

### Radiotherapy

Deep sedation or general anaesthesia was required for small children < 5 years who did not tolerate precision head mask fixation after a training period.

Twelve patients received FSRT as primary postoperative radiotherapy after surgical resection. In 6 patients irradiation of the posterior fossa was performed with a local boost to the tumor bed, and in 4 patients the tumor bed only was irradiated. In two patients, FSRT was applied as a boost in combination with CSI. A median dose of 54 Gy (range 46.8 Gy – 64 Gy) was prescribed in a median fractionation of 5 × 1.8 Gy per week for primary local RT using 6 MeV photons with a linear accelerator. For patients treated with a boost to the tumor bed, the posterior fossa was treated with a median dose of 45 Gy plus 9 Gy (median) to the tumor bed.

In 7 patients FSRT was performed as re-irradiation at the time of tumor progression. Patients had been treated previously with a median dose of 50 Gy (range 40 Gy – 55.2 Gy). The median time between primary irradiation and re-irradiation was 74 months (range 11 – 406 months). Imaging performed for treatment planning at the time of tumor progression was superimposed to the original treatment plans. All recurrent tumors were localized within the former high dose RT field.

We applied a median dose of 36 Gy (range 20 Gy – 60 Gy) in a median fractionation of 5 × 1.8 Gy (range 1.8 Gy – 2 Gy) for re-irradiation.

In seven patients CSI was performed. Five of these 7 patients received CSI as a component of primary RT for localized intracranial tumors, and were irradiated with FSRT at the time point of local tumor progression. Two patients received irradiation of the CSI together with FSRT because of spinal dissemination in the primary situation.

None of the patients received concomitant chemotherapy.

Three patients were treated according to studies performed by the German Pediatric Hematology and Oncology Society (GPOH) on multimodal treatment of Ependymomas (HIT) and received chemotherapy sequentially [24]. A group of three patients received chemotherapy for tumor progression, including vincristine, carboplatin, cisplatin, ifosfamide and etoposide.

FSRT was well tolerated by all patients and could be completed without interruptions due to side effects. No severe treatment related toxicity > CTC grade 2 for patients treated with FSRT could be observed.

Twelve patients remain alive after a median follow-up time of 32 months (7–185 months).

### Survival

**Figure 1:**
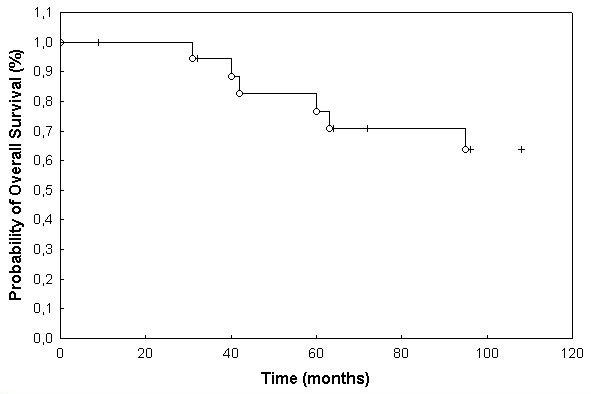
Overall survival of patients with histologically confirmed ependymoma treated with FSRT (77% at 3 years and 64% at 5 years).

**Figure 2:**
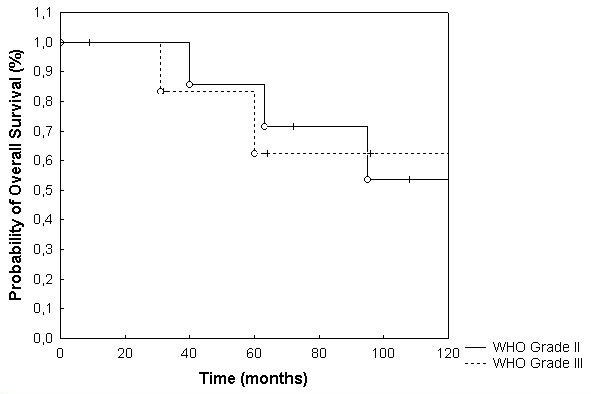
Overall survival broken down by histology (p = 0.92).

**Figure 3:**
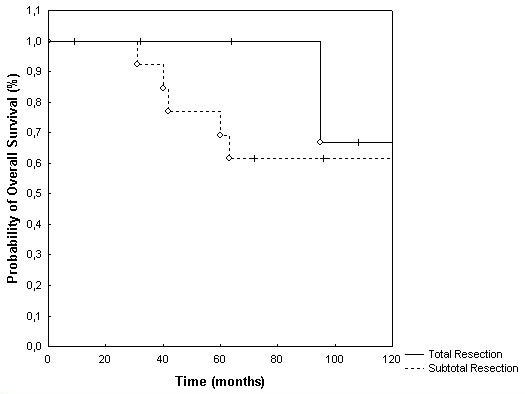
Overall survival broken down by the extent of neurosurgical resection (p = 0.54).

Overall survival for the total patient collective was 77% and 64% at 3 and 5 years, respectively (Fig. 1). With regard to histology, 3 and 5 years overall survival was 87% and 52% for grade II tumors and 64% and 64% for grade III tumors, respectively. This difference was not significant at p = 0.92 (p = 0.54; table 2; Fig. 2). Extent of surgical resection did not influence overall survival significantly (table 2, Fig. 3).

**Figure 4:**
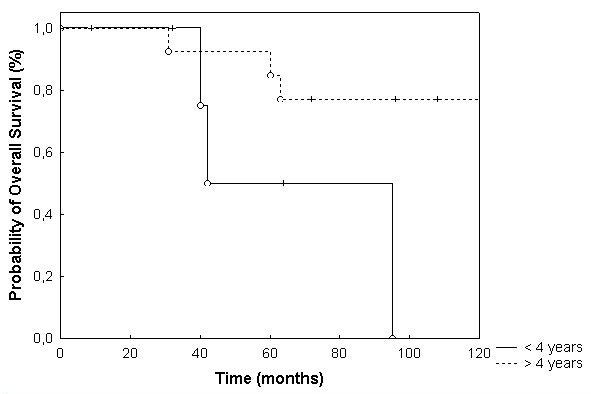
Overall survival broken down by age < 4 yrs. and ≥ 4 yrs (p = 0.04)

**Figure 5:**
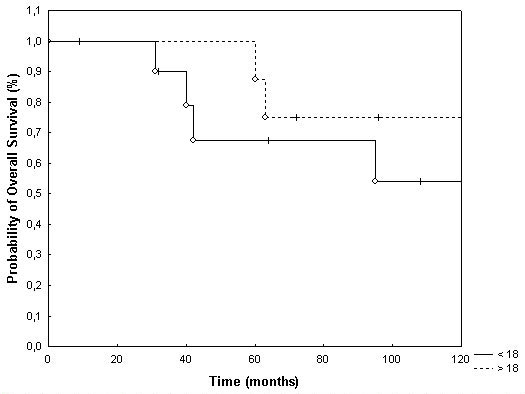
Overall survival broken down by age <18 yrs. and ≥ 18 yrs. (p = 0.39).

Overall survival in children < 4 years was 100% and 56% at 3 and 5 years, as opposed to 100% and 85% in patients ≥ 4 years of age (p = 0.04; Fig. 4). Age <18 vs. ≥ 18 also showed a trend towards a prolonged survival time in patients older than 18 years of age, which was however below the significance level (p = 0.39; Fig. 5). Potential prognostic factors evaluated are listed in table 2. Patients treated with FSRT for primary irradiation showed an overall survival of 100% and 78% at 3 and 5 years after irradiation of the posterior fossa with a boost to the tumor bed, and the group with treatment of the tumor bed only showed a survival rate of 100% at 5 years (p = 0.45). The two patients treated with FSRT and CSI as primary RT died of tumor progression after 28 and 48 months, respectively.

After re-irradiation with FSRT, survival rates of 83% and 50% at 3-and 5 years, respectively, were obtained.

### Local and distant control

**Figure 6:**
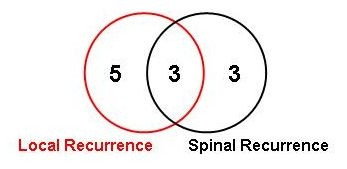
Eleven patients developed tumor progression after FSRT.

Eleven patients developed tumor progression after RT during follow-up: these events consisted of local recurrences in 5 patients, local recurrence and spinal dissemination in 3, and spinal metastases in 3 patients (Fig. 6). All local recurrences developed within the high dose area of the former RT-field. No new spinal recurrences developed after FSRT.

Progression-free survival rates after primary RT as compared to re-irradiation were 64% and 60% at 5 years, respectively, with local control rates at 3 and 5 years of 60%.

## Discussion

In recent years some progress has been made in the therapy of EPs. However, the prognosis for patients with EPs remains rather poor, and sound evidence regarding the main prognostic factors and the optimal therapeutic management strategy for EPs is still lacking.

The main primary treatment for EP is surgery, however, a complete removal of the tumor is rarely possible. Based on historical data and most recent series, there is a trend to consider the extent of surgery as a major prognostic factor [4,5]. Therefore, surgery is commonly the first procedure performed in patients with EP:

Survival rates for childhood EPs reported in the literature vary from 14% to 70% at 5 years [4,11,16,25-31]. In series including infratentorial lesions in children only, survival rates tend to be lower, whereas in series including adult EPs survival rates are much better [7,32]. In the present analysis survival rates at 5 and 10 years were 77% and 64%, respectively. In analogy to a variety of published data, age ≥ 18 seems to be associated with higher overall survival. Patients ≥ 18 yrs. showed 5 and 10 year overall survival rates of 88% and 74% as opposed to 68% and 54% in children < 18 years of age. Patients under 4 years of age demonstrated significantly worse overall survival times compared to patients ≥ 4 years of age (p = 0.04).

In a variety of studies, histologic grade of the tumor was also identified as a prognostic factor [16,26,33-38]. However, a number of studies including the present analysis did not reveal a significant influence of histologic grade on treatment outcome. Therefore, the possible correlation between tumor grade and survival is discussed controversially [4,7,11,16,29,34,39-41]. The different findings reported might be attributable to sample size, anatomic tumor location, variability in the definition of anaplasia, discrepancies in histological diagnosis, and the inclusion in some series of ependymoblastoma and subependymoma, which exhibit different biologic behaviour and should be analyzed separately [5,42-44].

Furthermore, there is still some controversy about the definition of the optimal treatment volume for EP. Large studies have shown no survival benefit for patients treated with whole-brain radiotherapy (WBRT) and irradiation of the craniospinal axis (CSA) as opposed to RT administered to smaller treatment volumes [25,33,45,46]. Kovalic reported a 10 year survival rate of 75% for patients treated with local RT as compared to 28% in patients treated with WBRT [45]. Furthermore, several investigations observed a lack of statistically significant improvement of outcome for patients treated with craniospinal irradiation [25,33,46-48]. However, RT of the CSA was frequently used in the treatment of high-grade ependymomas due to their anticipated higher risk of metastatic cerebrospinal fluid (CSF) seeding [15,25,34,49,50].

However, in several series the most common area of recurrence was locally, within the former RT field [25,33,46-48]. To improve local control, high local doses to the defined target volume are essential. In the past, a number of studies have proven a significant correlation between adequate RT dose and local control. An RT dose between 50 Gy and 60 Gy has been shown to be effective and high local control rates can be achieved, with 54 Gy being considered the standard dose and higher doses being prescribed in investigational studies. Garrett et al. showed overall survival rates of 14% in patients treated with less than 45 Gy, and a survival rate of 50% in patients treated with more than 45 Gy [16]. Goldwein et al reported similar results, with overall survival rates of 18% in patients treated with less than 45 Gy as compared to survival rates of 51% in patients treated with doses ≥ 45 Gy [17]. Therefore, based on the known dose-response relationship of EP, an improvement in RT techniques might help improve local control, enabling escalation of local RT doses while minimizing irradiation of surrounding normal tissue.

This might be achieved by applying modern RT techniques such as stereotactic radiosurgery (SRS) and fractionated stereotactic radiotherapy (FSRT). High precision radiotherapy allows the application of high doses to a defined target volume while sparing normal tissue [19]. In the past, high precision radiotherapy techniques were not applied routinely in the management of EP. With SRS, high local doses are applied in a single fraction[19]. SRS has been applied in a small number of studies in patients with EP: Hodgson et al. published the largest series applying SRS in patients with EP, reporting a 3-year progression-free survival rate of 22% in 28 paediatric patients. Jawahar et al. report the outcome of 22 patients treated with SRS for anaplastic EP, with a 5-year actuarial progression-free survival rate of 32.4%. A recent study published by Mansur et al. showed a 3-year relapse free survival rate of 55.6%, and 3-year overall survival rate of 71.1% in nine patients with EP treated with SRS [51,52]. However, SRS is limited to small targets, and therefore this technique is preferred especially in small recurrent lesions or as a boost modality for macroscopic residuals in combination with external beam RT to the tumor bed [51]. With fractionated stereotactic radiotherapy (FSRT), larger target areas can be treated while exploiting the biological effect of fractionation. However, in the literature little data on FSRT in patients with EP can be found. The results of the present study support the idea, that FSRT can be applied safely in patients with primary or recurrent EP. Overall survival and local control rates are comparable to data found in the literature and show that FSRT can help improving local control without leading to an increased rate of marginal recurrences.

The role of chemotherapy (CHT) in the management of EP still remains unclear [27,53]. In general, it would be reserved for patients after failure of previously performed surgery and RT [35,54,55]. The German Society of Paediatric Hematology and Oncology is conducting a large study for children with EP, investigating concomitant chemotherapy with vincristine for WHO grade II and III EP in children between age 4 and 21 with persisting tumor after initial RT. Adjuvant chemotherapy with cisplatin, vincristine, carboplatin and VP16 is recommended for Grade III EP. In children younger than age of 4, chemotherapy including cyclophosphamide, vincristine and methotrexate is recommended after surgery [24]. Needle et al. report a benefit in overall survival for patients treated with carboplatin, vincristine, ifosfamide and etoposide [56]. In spite of incomplete surgical resection, progression-free survival after 5 years was 80%. However, hyperfractionated RT was also included into this regime. Kühl et al. could demonstrate a rate of 55% of partial or complete remissions after postoperative CHT before the initiation of RT in patients with anaplastic EP [57]. In a study performed by the Royal Marsden Hospital, London, 10-year overall survival was 54% in patients treated with CCNU as adjuvant CHT as opposed to 34% in patients treated with RT only [14]. In children < 2–3 years of age, the up-front application of CHT can help delay the need to initiate RT [46,58-62]. In the present analysis, three children were treated according to studies performed by the GPOH on multimodality treatment of EP (HIT) and received chemotherapy sequentially [24]. A group of three patients received chemotherapy for tumor progression, including vincristine, carboplatin, cisplatine, ifosfamide and etoposide.

The results of large randomized trials evaluating the role of adjuvant CHT are anticipated.

## Conclusion

Until now, no prospective trials including FSRT have been performed involving children and adults with EP. Therefore, the current analysis allows us to conclude that FSRT can be applied in patients with EP without an increased risk for marginal failures. The main advantage of high precision RT, i.e. the local application of high doses while sparing normal tissue, is especially helpful in smaller children with respect to early and late neurocognitive sequelae and growth deficits caused by irradiation of normal tissue in young children. FSRT is a useful tool for reirradiation of local recurrences and yields good local control rates in a considerable proportion of patients.

## Competing interests

The author(s) declare that they have no competing interests.

## Authors' contributions

SEC, CT, JD and DSE treated the patients, performed radiotherapy treatment planning, collected the clinical data useful for the analysis as well as wrote and revised the article critically for important intellectual content. All authors read and approved the final manuscript.

## Pre-publication history

The pre-publication history for this paper can be accessed here:



## Figures and Tables

**Table 1:** Patient and tumor characteristics for the entire study population (n = 19)

**Characteristic**	**No. of patients (%)**
age	
< 4	5 (26%)
≥ 4	14 (74%)
gender	
female	9 (47%)
male	10 (53%)
histology	
II	12 (63%)
III	7 (37%)
tumor site	
supratentorial	4 (21%)
infratentorial	15 (79%)
extent of surgery	
total resection	5 (26%)
subtotal resection	14 (74%)

**Table 2:** Univariate Analysis of Overall Survival among patients with Grade 2 and Grade Ependymomas (n = 19) treated with Fractionated Stereotactic Radiotherapy (FSRT).

Variable	5 yrs. Survival rate	10 yrs. Survival rate	P (Cox regression)
all patients	77%	64%	
age			
< 4	56%	18%	0.04
≥ 4	85%	76%	
gender			
female	76%	61%	0.92
male	78%	66%	
histology			
II	87%	52%	0.92
III	64%	64%	
tumor site			
supratentorial	100%	0%	0.44
infratentorial	72%	55%	
extent of surgery			
total resection	100%	67%	0.54
subtotal resection	70%	62%	
primary radiotherapy			
FSRT+CSI	-	-	0.45
posterior fossa + boost	78%	78%	
tumor bed	100%	100%	
